# A narrative review of minimally invasive techniques in restorative dentistry

**DOI:** 10.1016/j.sdentj.2023.11.005

**Published:** 2023-11-04

**Authors:** Yasir Alyahya

**Affiliations:** Department of Conservative Dental Science, College of Dentistry, Qassim University, Qassim, Saudi Arabia

**Keywords:** Minimally invasive techniques, Restorative dentistry, Review, Evidence-based studies, Atraumatic restorative treatment, Minimal intervention dentistry

## Abstract

This narrative review aimed to provide a comprehensive overview of minimal invasive dentistry (MID) by synthesizing relevant articles obtained from various sources, including electronic databases such as PubMed, SCOPUS, EMBASE, the COCHRANE library, and Science Direct, as well as through manual searches of cross-references and textbooks. The search employed MeSH terms and keywords related to MID, such as “minimally invasive dentistry,” “atraumatic restorative treatment (ART),” “MID,” and “minimum intervention dentistry.” The inclusion criterion was English-language articles published between the years 2000 and June 2023 that aligned with the study objectives. After a thorough assessment of the included articles, 34 high-quality articles were selected for this review. The selected articles elucidate the characteristics of MID, the application of the ART, and the principles of minimum intervention in dentistry. Animal-based studies and narrative reviews on MID were excluded from the analysis. This narrative review serves as a valuable resource for dental professionals, researchers, and educators interested in staying abreast of the latest developments and evidence in the field of MID.

## 1 Introduction

Restorative dentistry plays a pivotal role in preserving and enhancing oral health by repairing damaged teeth and restoring functionality and aesthetics. In recent years, there has been a paradigm shift in dental practice towards minimally invasive techniques that aim to conserve healthy tooth structures while achieving optimal treatment outcomes (Murdoch-Kinch and McLean, 2003). This narrative review analyzes minimally invasive techniques in restorative dentistry and explores their advantages, challenges, and potential applications.

The traditional approach to restorative dentistry often involves the removal of substantial tooth structures to accommodate restorations such as dental crowns or bridges (Ericson, 2007). However, this approach has several drawbacks, including loss of healthy tooth material, increased susceptibility to future complications, and prolonged treatment time. In response to these limitations, minimally invasive techniques have emerged as progressive alternatives, promoting conservative approaches that prioritize the preservation of the natural tooth structure (Ericson, 2007).

This review examines various minimally invasive techniques employed in restorative dentistry, including adhesive dentistry, composite resin restoration, and biomimetic dentistry. By utilizing state-of-the-art materials and innovative bonding protocols, these techniques aim to mimic the natural structure and function of teeth while minimizing invasiveness. We explored scientific evidence supporting the efficacy and longevity of these approaches, as well as their potential impact on patient satisfaction and oral health.

Furthermore, this review highlights the challenges associated with implementing minimally invasive techniques in everyday dental practice, including case selection, proper diagnosis, and the development of precise treatment plans. By critically evaluating the available literature, we aimed to provide a comprehensive overview of the current state of minimally invasive restorative dentistry and its implications in clinical practice. Hence, this review aimed to elucidate the potential benefits and limitations of minimally invasive techniques in restorative dentistry, facilitate informed decision-making for dental practitioners, and foster a patient-centered approach that prioritizes conservative and sustainable dental care.

## 2 Materials and methodology

**Fig. 1:**
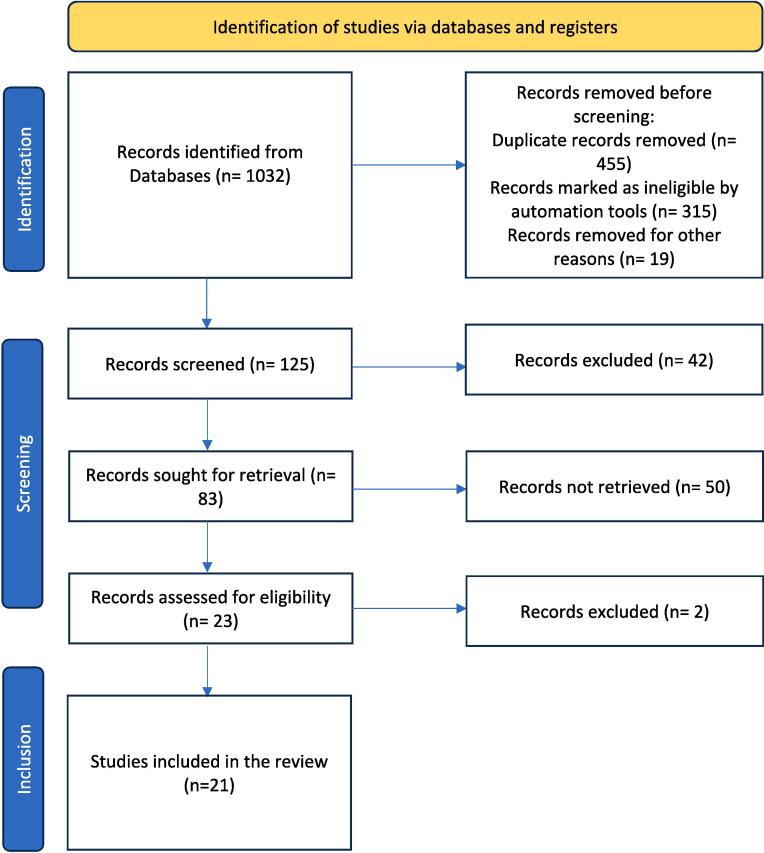
Flowchart showing the step-by-step identification of the studies via databases.

To conduct a literature survey on minimally invasive dentistry, a search was conducted in June 2023 across various electronic databases, including PubMed, SCOPUS, EMBASE, COCHRANE Library, and Science Direct. The search utilized MeSH terms/keywords such as “minimal invasive dentistry,” “atraumatic restorative treatment (ART),” “MID,” and “minimum intervention dentistry.” In addition to the electronic searches, cross-references and textbooks were manually searched for relevant articles. Articles published in English between the year 2000 and June 2023 that fulfilled the study’s objectives were included. The article selection process involved assessing the inclusion and exclusion criteria and conducting a quality assessment. Of the initial 1032 identified articles, 121 were selected based on their titles and abstracts. An additional four articles were obtained through a manual search, resulting in a total of 83 articles. After evaluating the full texts and applying the inclusion and exclusion criteria, 21 articles were chosen for the review, meeting the study's criteria. Animal studies and narrative reviews of MID were excluded from the selection process (Fig. 1).

## 3 Overview of restorative dentistry

Restorative dentistry is a specialized branch of dentistry that focuses on diagnosing, preventing, and treating dental diseases and conditions that affect tooth functionality and appearance (White and Eakle, 2000). Restorative dentistry primarily aims to restore damaged or missing teeth, enabling patients to regain their oral health, function, and esthetics. This field encompasses a wide range of treatments, including dental fillings, crowns, bridges, implants, and dentures (Christensen, 2005).

Traditionally, restorative dentistry involves the removal of a significant amount of healthy tooth structure to accommodate restorations. For instance, dental crowns and bridges require a reduction of tooth enamel, leading to the loss of valuable natural tooth material. However, this approach has several challenges and drawbacks (Brostek et al., 2006).

One of the main challenges of traditional restorative techniques is the increased risk of complications (Showkat et al., 2020). The removal of a substantial portion of the tooth structure weakens the tooth, making it more susceptible to fractures or further decay over time (Gutmann, 2013). Furthermore, the extensive tooth preparation required in the traditional approaches often results in prolonged treatment times and increased patient discomfort (Neena et al., 2015). Additionally, traditional approaches in restorative dentistry often result in poor esthetic outcomes. The materials used, such as metal-based restorations, lack the natural appearance of teeth and compromise the overall smile esthetics. This limitation affects patient satisfaction and self-confidence (Katz et al., 2013).

Moreover, the traditional approach to restorative dentistry does not prioritize the preservation of the natural tooth structure, leading to unnecessary loss of healthy teeth. This approach conflicts with the principles of conservative dentistry and preventive care, which emphasize minimal intervention and preservation of tooth structure whenever possible (Kumar and Neha, 2013). Minimally invasive dentistry aims to conserve as much healthy tooth structure as possible while achieving optimal treatment outcomes in restorative dentistry. It emphasizes the preservation of natural tooth materials and focuses on preventive measures to maintain oral health. The principles of minimally invasive dentistry include early detection, intervention, precise diagnosis, conservative treatment planning, and the use of advanced materials and techniques (Kielbassa et al., 2009).

Minimally invasive restorative dentistry techniques offer numerous advantages over traditional approaches. These include preserving a healthy tooth structure, prioritizing the conservation of natural tooth material, and reducing the need for extensive tooth preparation (Weisrock et al., 2011). This approach helps to maintain the structural integrity of the tooth. Additionally, minimally invasive techniques minimize patient discomfort during treatment and promote faster recovery (Shah et al., 2016). Furthermore, advanced materials such as tooth-colored composite resins and ceramic restorations closely resemble natural teeth, resulting in improved esthetic outcomes (LeSage, 2009). Finally, by preserving more tooth structure and utilizing adhesive bonding techniques, minimally invasive approaches contribute to improved long-term stability and durability of restorations (Elnawam et al., 2022).

## 4 Recent advances in minimally invasive techniques

### 4.1 Microabrasion

This is a minimally invasive technique used to remove superficial enamel discolorations and defects. It involves mechanical abrasion of the outer enamel layer using a combination of mild abrasive agents and a high-speed handpiece (Rayapudi and Usha, 2018). This procedure is particularly effective for addressing white or brown demineralized spots, fluorosis stains, and mild enamel hypoplasia. Microabrasion can significantly improve the esthetics of affected teeth without the need for invasive interventions or extensive tooth preparation. It is often combined with other minimally invasive techniques such as enamel bonding or bleaching to achieve optimal results (Kornblit et al., 2008).

### 4.2 Resin infiltration

Resin infiltration is used to treat incipient or non-cavitated carious lesions, particularly those affecting the enamel. This involves the infiltration of a low-viscosity resin into the porous enamel structure, which effectively arrests the progression of the lesion and improves its appearance. The resin infiltrant fills the voids within the enamel, reinforcing its structural integrity, and masking the discoloration caused by carious lesions. This minimally invasive approach avoids the removal of healthy tooth structures and is an effective preventive measure for halting the progression of early caries. Resin infiltration is often used in combination with other minimally invasive restorative techniques to provide comprehensive care for dental caries (Mirsiaghi et al., 2018).

### 4.3 Bleaching

Tooth bleaching is a common minimally invasive technique used to address tooth discoloration and enhance smile esthetics. It involves the application of bleaching agents such as hydrogen peroxide or carbamide peroxide to remove intrinsic and extrinsic stains from the tooth structure. Bleaching can be performed in-office under professional supervision or at home using customized trays and bleaching gels. This technique is effective for improving the shade and brightness of teeth without the need for invasive procedures. It is particularly beneficial in patients with tooth discoloration caused by aging, tobacco use, or certain dietary habits. Bleaching offers a conservative and cost-effective approach to enhance smile esthetics and can be combined with other minimally invasive procedures, such as microabrasion or composite resin restorations, for comprehensive results (Prabhakar et al., 2015).

### 4.4 Digital dentistry and Computer-aided design/computer-aided manufacturing (CAD/CAM) technology

CAD/CAM enables the precise fabrication of restorations, reducing the need for traditional impressions and facilitating minimally invasive treatment planning (Kumar et al., 2021).

### 4.5 Biomimetic approaches

Biomimetic dentistry aims to mimic the natural structure and function of teeth using materials and techniques that closely resemble natural tooth properties, resulting in enhanced longevity and esthetics (Da Mata et al., 2015).

### 4.6 Laser-assisted techniques

Dental lasers provide precise and minimally invasive treatment options for various procedures, including cavity preparation, soft tissue management, and tooth whitening (Banerjee, 2015).

### 4.7 Nanotechnology in restorative materials

**Fig. 2:**
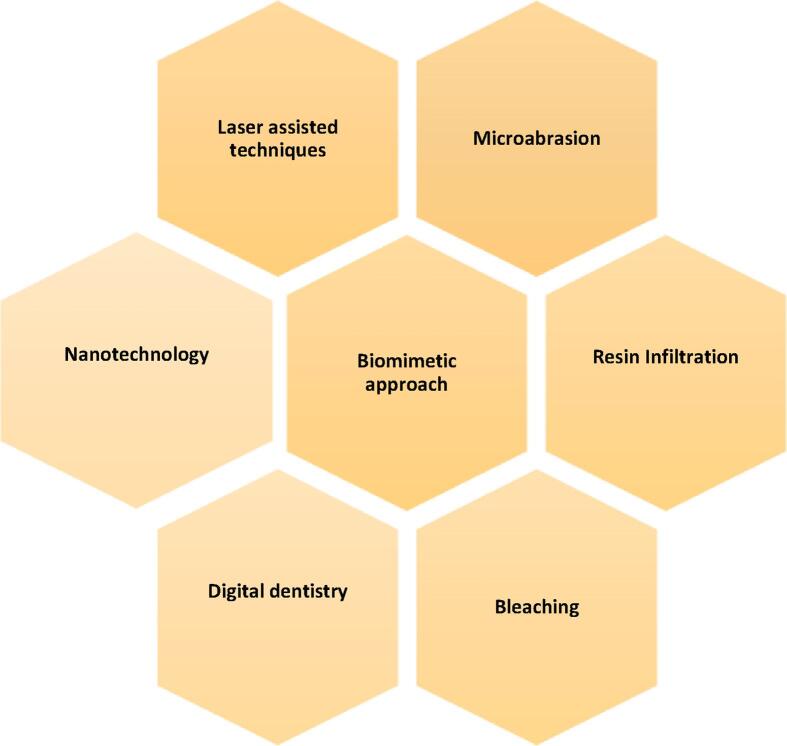
Minimally invasive techniques in restorative dentistry.

Nanomaterials offer improved physical and mechanical properties, enabling the creation of stronger and more esthetic restorations with minimal tooth preparation (Tumenas et al., 2014) (Fig. 2).

Recent advances in minimally invasive techniques continue to revolutionize restorative dentistry by providing more efficient, patient-centered, and sustainable treatment options.

## 5 Clinical applications and evidence of minimally invasive techniques

### 5.1 Clinical indications for minimally invasive techniques

Minimally invasive restorative dentistry techniques have a broad range of clinical indications. They can be used to treat various conditions, including.

#### 5.1.1 Small-to-moderate-sized carious lesions

Minimally invasive techniques, such as composite resin restorations, are well suited for the conservative repair of small-to-moderate-sized cavities, preserving as much healthy tooth structure as possible (Newton and Asimakopoulou, 2017).

#### 5.1.2 Tooth wear

Minimally invasive approaches can effectively restore teeth affected by attrition, abrasion, or erosion, thereby addressing functional and esthetic concerns while minimizing tooth preparation (Alam et al., 2021).

#### 5.1.3 Enamel defects and discolorations

Techniques such as veneers and bonding are used to correct enamel defects such as enamel hypoplasia or fluorosis and to mask discoloration, providing a more esthetic appearance (Creugers, 2003).

#### 5.1.4 Fractured or chipped teeth

Minimally invasive techniques offer effective solutions for restoring fractured or chipped teeth by utilizing adhesive bonding and composite resin materials to achieve functional and natural-looking results (Weinstein et al., 2021).

### 5.2 Long-term clinical outcomes and success rates

Numerous studies have investigated the long-term clinical outcomes and success rates of minimally invasive techniques in restorative dentistry Nizami et al., 2022, Kaidonis et al., 2013). These studies have consistently shown favorable results. For example, long-term follow-up studies on composite resin restorations have demonstrated high survival rates, with success rates ranging from 80 % to 95 % after 5 years to 10 years (Hochman, 2006). Ceramic inlays and onlays exhibit excellent longevity and clinical performance. Long-term studies have reported survival rates of approximately 90 % after 10 years, with minimal complications or failures (Zhang et al., 2015).

Moreover, veneer and bonding techniques have shown satisfactory long-term outcomes, with high patient satisfaction and minimal restoration failure. Proper case selection, meticulous adhesive bonding, and regular maintenance contribute to the longevity and success of minimally invasive restorations.

### 5.3 Patient satisfaction and acceptance

Patient satisfaction and acceptance are essential factors in evaluating the success of minimally invasive techniques. Several studies have indicated high levels of patient satisfaction and acceptance of these approaches (Patri and Sahu, 2017). Patients appreciate the preservation of their natural tooth structure, the minimally invasive nature of the procedures, and the improved esthetics achieved through tooth-colored restorations.

Minimally invasive techniques also tend to result in lower postoperative sensitivity and discomfort compared to traditional approaches. Patients experience shorter treatment times, a reduced need for anesthesia, and faster recovery, leading to higher levels of satisfaction.

### 5.4 Comparative studies with traditional approaches

Comparative studies have been conducted to evaluate the effectiveness and advantages of minimally invasive techniques compared with traditional approaches (Banerjee, 2013, Nový and Fuller, 2008). These studies have consistently shown that minimally invasive techniques provide comparable or superior outcomes, with several advantages over traditional approaches. For example, studies comparing composite resin restorations with traditional amalgam restorations have demonstrated similar or better longevity, improved esthetics, and reduced risk of tooth fracture are observed with composite resin restorations.

Studies have shown that ceramic restorations offer superior esthetics, longevity, and biocompatibility than those offered by traditional metal-based restorations.

Comparative studies evaluating the outcomes of minimally invasive veneers and bonding techniques have reported favorable results compared to more invasive procedures, such as full crowns or extensive tooth preparation.

## 6 Challenges and limitations of minimally invasive techniques in restorative dentistry

Although minimally invasive techniques in restorative dentistry offer numerous advantages, they encounter certain challenges and limitations that must be considered. Understanding these challenges is crucial for dental practitioners to provide effective and appropriate patient care.

### 6.1 Case selection and complexity

One of the primary challenges in implementing minimally invasive techniques is accurate case selection and assessment of the complexity of the dental condition. Minimally invasive approaches are not suitable for all cases, and certain situations may require more extensive restorative procedures. In complex cases involving extensive tooth damage, a compromised tooth structure, or multi-tooth restorations, a more invasive approach may be necessary to achieve optimal outcomes. Dental practitioners must have a thorough understanding of case selection criteria and treatment planning to ensure the appropriate application of minimally invasive techniques (Attik et al., 2022).

### 6.2 Operator skill and learning curve

Minimally invasive techniques often require specialized skills and expertise. Dental practitioners must possess the necessary knowledge, training, and experience to perform these techniques effectively. The learning curve associated with adopting minimally invasive approaches can be steep, and it may take time and practice to master the precise techniques and use advanced materials. Lack of operator skill or inadequate training can lead to suboptimal results, compromised longevity of restorations, or an increased risk of complications. Continued professional development and hands-on training are essential to overcome this limitation and ensure the successful implementation of minimally invasive techniques (Albeshir et al., 2022).

### 6.3 Material limitations and durability

The selection and limitations of restorative materials play crucial roles in the success and longevity of minimally invasive restorations. Although significant advancements have been made in composite resin materials and ceramics, they still have limitations in terms of durability and long-term performance. For instance, composite resins may be susceptible to wear, staining, or chipping over time, and require regular maintenance or replacement. Ceramics, although more resistant to wear and discoloration, may be more brittle and prone to fracture than natural tooth structures. It is important to consider specific material properties, patient factors, and occlusal forces when choosing and using restorative materials to ensure the longevity and success of minimally invasive restorations (Okuda, 2013).

### 6.4 Cost considerations

Another limitation of minimally invasive restorative dentistry techniques is the high cost associated with the advanced materials, equipment, and technology. High-quality materials and equipment may come with a higher price tag, which can affect the overall treatment cost. In addition, the learning curve and training required to implement these techniques effectively can incur additional costs. Although the long-term benefits and advantages of minimally invasive approaches are well-established, cost considerations may limit access and utilization for some patients. Dental practitioners must consider the financial implications and communicate transparently with their patients to provide appropriate treatment options based on individual needs and budgets (Oliveira et al., 2016).

## 7 Future directions in minimally invasive techniques in restorative dentistry

### 7.1 Potential advancements and innovations

The field of minimally invasive dentistry is constantly evolving and several potential advancements and innovations are awaited. These include:

#### 7.1.1 Advanced restorative materials

Ongoing research is focused on developing new restorative materials with enhanced durability, esthetics, and biocompatibility. Biomimetic materials that closely mimic the natural properties of teeth, such as bioactive composites and smart materials, have been explored for their potential use in minimally invasive restorations (Whitehouse, 2004).

#### 7.1.2 Digital Dentistry and three-dimensional (3D) printing

The integration of digital dentistry and 3D printing technology offers exciting possibilities for minimally invasive dentistry. CAD and CAM systems combined with 3D printing enable the precise fabrication of restorations and improve accuracy, efficiency, and customization (Cullum and Deporter, 2015).

#### 7.1.3 Regenerative approaches

Regenerative techniques such as stem cell therapies and tissue engineering regenerate damaged or lost dental tissues. These approaches may revolutionize restorative dentistry by promoting natural healing and regeneration of tooth structures, thereby reducing the need for invasive interventions (Manaia et al., 2021).

#### 7.1.4 Minimally invasive implant dentistry

Advancements in implant dentistry have focused on the development of minimally invasive techniques for implant placement and restoration. Guided implant surgery, immediate implant loading, and innovative implant materials are being explored to optimize treatment outcomes while minimizing invasiveness (Nguyen et al., 2022).

## 8 Importance of continuing research in minimally invasive techniques

Continued research is vital for the advancement and refinement of minimally invasive restorative dentistry. Research efforts should focus on the following aspects.

### 8.1 Long-term clinical studies

Long-term clinical studies will provide valuable data on the durability, success rates, and patient satisfaction associated with minimally invasive techniques. This will help to establish evidence-based guidelines and protocols for effective implementation (Arrow et al., 2018).

### 8.2 Comparative effectiveness studies

Comparative studies comparing the outcomes of minimally invasive techniques with those of traditional approaches are essential to establish the superiority, benefits, and cost-effectiveness of these techniques. These studies provide further evidence for the adoption and integration of minimally invasive approaches in routine clinical practice (Maldupa et al., 2022).

### 8.3 Material development and testing

Research should focus on the development and testing of new materials with improved properties, longevity, and esthetics for minimally invasive restorations. This includes investigating the behavior of materials under different oral conditions and assessing their biocompatibility, durability, and resistance to wear and discoloration (Hayes et al., 2014).

## 9 Conclusion

Minimally invasive techniques in restorative dentistry have emerged as an important approach to preserve natural tooth structure, achieve optimal treatment outcomes, and improve patient satisfaction. The future of minimally invasive dentistry is promising with potential advancements in materials, technology, and regenerative approaches.

Continued research is vital for the further refinement and validation of these techniques. Long-term clinical studies, comparative effectiveness studies, and material development research are crucial for establishing evidence-based protocols, enhancing treatment outcomes, and expanding the range of applications of minimally invasive approaches.

By embracing the principles of minimally invasive dentistry and integrating advancements in technology and materials, dental practitioners can provide patients with conservative, effective, and patient-centered care. This leads to improved oral health outcomes, enhanced esthetics, and increased patient satisfaction.

## Declaration of competing interest

The authors declare that they have no known competing financial interests or personal relationships that could have appeared to influence the work reported in this paper.
